# Neurodegenerative Disease Treatment Drug PBT2 Breaks Intrinsic Polymyxin Resistance in Gram-Positive Bacteria

**DOI:** 10.3390/antibiotics11040449

**Published:** 2022-03-25

**Authors:** David M. P. De Oliveira, Bernhard Keller, Andrew J. Hayes, Cheryl-Lynn Y. Ong, Nichaela Harbison-Price, Ibrahim M. El-Deeb, Gen Li, Nadia Keller, Lisa Bohlmann, Stephan Brouwer, Andrew G. Turner, Amanda J. Cork, Thomas R. Jones, David L. Paterson, Alastair G. McEwan, Mark R. Davies, Christopher A. McDevitt, Mark von Itzstein, Mark J. Walker

**Affiliations:** 1 Australian Infectious Diseases Research Centre, School of Chemistry and Molecular Biosciences, The University of Queensland, Brisbane, QLD 4072, Australia; d.deoliveira@uq.edu.au (D.M.P.D.O.); keller.ber@protonmail.ch (B.K.); cheryl-lynn.ong@qimrberghofer.edu.au (C.-L.Y.O.); n.harbisonprice@uq.edu.au (N.H.-P.); tom.li@uq.edu.au (G.L.); nadia.keller@gmail.com (N.K.); lisa_bo1@yahoo.de (L.B.); s.brouwer@uq.edu.au (S.B.); a.turner@uq.edu.au (A.G.T.); a.cork@uq.edu.au (A.J.C.); mcewan@uq.edu.au (A.G.M.); 2 Department of Microbiology and Immunology, Peter Doherty Institute for Infection and Immunity, The University of Melbourne, Melbourne, VIC 3000, Australia; andrew.hayes@unimelb.edu.au (A.J.H.); mark.davies1@unimelb.edu.au (M.R.D.); christopher.mcdevitt@unimelb.edu.au (C.A.M.); 3 Institute for Glycomics, Griffith University, Gold Coast, QLD 4222, Australia; i.el-deeb@griffith.edu.au (I.M.E.-D.); m.vonitzstein@griffith.edu.au (M.v.I.); 4 School of Earth and Environmental Sciences, The University of Queensland, Brisbane, QLD 4072, Australia; t.jones@uq.edu.au; 5 Australian Infectious Diseases Research Centre, UQ Centre for Clinical Research, The University of Queensland, Brisbane, QLD 4006, Australia; david.antibiotics@gmail.com

**Keywords:** antimicrobial resistance, polymyxins, PBT2, ionophores, Gram-positive bacteria

## Abstract

Gram-positive bacteria do not produce lipopolysaccharide as a cell wall component. As such, the polymyxin class of antibiotics, which exert bactericidal activity against Gram-negative pathogens, are ineffective against Gram-positive bacteria. The safe-for-human-use hydroxyquinoline analog ionophore PBT2 has been previously shown to break polymyxin resistance in Gram-negative bacteria, independent of the lipopolysaccharide modification pathways that confer polymyxin resistance. Here, in combination with zinc, PBT2 was shown to break intrinsic polymyxin resistance in *Streptococcus pyogenes* (Group A *Streptococcus*; GAS), *Staphylococcus aureus* (including methicillin-resistant *S. aureus*), and vancomycin-resistant *Enterococcus faecium*. Using the globally disseminated M1T1 GAS strain 5448 as a proof of principle model, colistin in the presence of PBT2 + zinc was shown to be bactericidal in activity. Any resistance that did arise imposed a substantial fitness cost. PBT2 + zinc dysregulated GAS metal ion homeostasis, notably decreasing the cellular manganese content. Using a murine model of wound infection, PBT2 in combination with zinc and colistin proved an efficacious treatment against streptococcal skin infection. These findings provide a foundation from which to investigate the utility of PBT2 and next-generation polymyxin antibiotics for the treatment of Gram-positive bacterial infections.

## 1. Introduction

Polymyxin antimicrobials are clinically employed as last-resort therapies for infections caused by multidrug-resistant *Pseudomonas aeruginosa* and carbapenemase-producing *Enterobacterales* [1]. The specificity of polymyxin antibiotics for Gram-negative bacteria arises from their targeting of the lipopolysaccharide (LPS) structure present on the outer- and cytoplasmic membranes [2,3]. The lack of LPS in Gram-positive bacteria explains their lack of susceptibility to this class of antimicrobial. Previously, we reported that the 8-hydroxyquinoline analog PBT2 (2-(dimethylamino) methyl-5,7-dichloro-8-hydroxyquinoline) [4,5] can break polymyxin resistance in a range of World Health Organization defined, critical Gram-negative pathogens, independent of the LPS modification pathways that confer polymyxin resistance [6].

In both community and clinical settings, Gram-positive bacteria, such as *Enterococcus faecium*, *Staphylococcus aureus*, and *Streptococcus pyogenes*, are prominent contributors of morbidity and mortality [1,7]. For *E. faecium*, hospital-adapted lineages have become increasingly resistant to vancomycin (vancomycin-resistant *E. faecium*; VRE) [8,9]. Compared to infection with vancomycin-susceptible *E. faecium*, infection with VRE is associated with excess mortality, duration of hospital admission, and treatment costs [10,11,12]. Equally, the presence of methicillin resistance in *S. aureus* bloodstream infection has been independently associated with a 20% increase in mortality [13,14]. For *S. pyogenes* (Group A *Streptococcus*; GAS), non-invasive states of GAS disease (i.e., pharyngitis, scarlet fever, and impetigo) [15] are typically self-limiting; treatment with either penicillin or amoxicillin are commonly employed strategies used to decrease the duration of non-invasive GAS illness [16]. However, for invasive GAS infections, disease states such as necrotizing fasciitis and streptococcal toxic shock syndrome, frequently result in patient intensive care admission, and are associated with poor patient outcome [7].

Here, we demonstrate that, in the presence of zinc, PBT2 renders Gram-positive pathogens susceptible to polymyxin antibiotics. Using a murine wound infection model, PBT2 in combination with zinc and colistin proved efficacious against GAS skin infection. PBT2 is an orally bioavailable hydroxyquinoline ionophore that facilitates the transfer of zinc across biological membranes. PBT2 has completed phase 2 clinical trials for neurodegenerative conditions, in which once daily oral dosing of 250 mg was generally safe and well tolerated when administered for up to 12 months (EURO, REACH, and IMAGINE clinical trials) [5,17,18]. This study describes a conceptual approach to exploit the utility of next-generation polymyxins as potential therapies for antimicrobial resistant Gram-positive bacterial infections.

## 2. Results

PBT2 has been previously shown to break polymyxin resistance in Gram-negative bacterial pathogens, independent of the LPS modifications that confer polymyxin resistance [6]. To investigate whether PBT2 could break intrinsic polymyxin resistance in Gram-positive pathogens, assorted strains of GAS, *Staphylococcus aureus*, and *Enterococcus* were employed for antimicrobial sensitivity testing in accordance with the Clinical and Laboratory Standards Institute (CLSI) and the European Committee on Antimicrobial Susceptibility Testing (EUCAST) guidelines [19,20]. The assemblage of respective bacterial strains included the globally disseminated clonal M1T1 GAS strain 5448 [21], methicillin-resistant *Staphylococcus aureus* strain USA300 [22], and vancomycin-resistant *Enterococcus faecium* strain RBWH1 [23]. In the presence of antibiotic alone, all GAS, *S. aureus*, and *Enterococcus* strains were shown to be resistant to colistin (Table 1). The addition of zinc alone did not alter the susceptibility profiles to colistin. In the presence of PBT2, all strains showed a moderate reduction in the minimum inhibitory concentration (MIC) for colistin. In the presence of PBT2 + zinc, all GAS, *S. aureus*, and *Enterococcus* strains showed a further reduction in the MIC for colistin. Comparison of the resultant MICs with EUCAST-defined polymyxin breakpoints for Gram-negative bacteria (≤2 µg/mL) [20] indicated that the combination treatment of PBT2 + zinc rendered each strain of GAS, *S. aureus*, and *Enterococcus* susceptible to colistin (Table 1).

**Figure 1:**
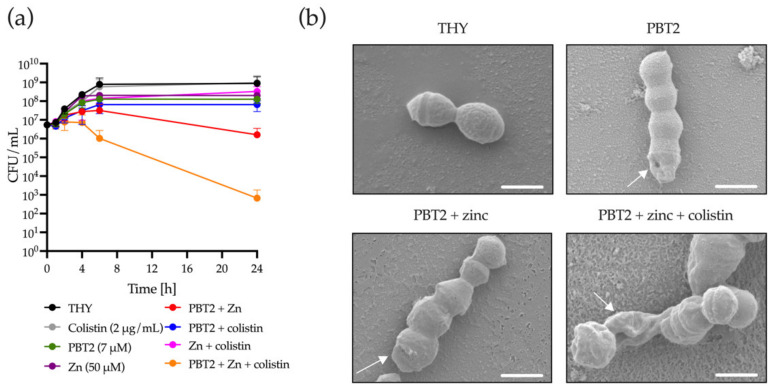
PBT2 + zinc in combination with colistin induces a bactericidal effect against GAS strain 5448. (**a**) Time-kill curves for GAS strain 5448 in Todd–Hewitt broth supplemented with 1% yeast extract (THY). GAS strain 5448 was treated with or without PBT2, zinc, and colistin. Error bars indicate standard deviation from three biological replicates. CFU, colony forming unit. (**b**) Scanning electron microscopy images of GAS strain 5448 grown in THY media, or in THY media supplemented with combinations of PBT2 (7 µM), zinc (50 µM), and colistin (2 µg/mL) for 24 h at 37 °C. White scale bars = 1 µm. Arrows indicate membrane indentations and/or membrane ruffling.

Using the GAS strain 5448 as a proof of principle model, the combination of PBT2 + zinc in the presence of colistin was observed to be bactericidal, indicated by >3-log_10_ reduction in viable bacteria over 24 h (Figure 1a). Microscopy analysis of GAS strain 5448 identified that PBT2 alone altered bacterial cell morphology, as evidenced through bacterial membrane indentations. Membrane ruffling and apparent cell rupture was observed for GAS treated with PBT2 + zinc and colistin (Figure 1b). Zinc or colistin alone, or in combination, had no observable effect on bacterial cell morphology (Figure 1b and Figure S1).

**Figure 2:**
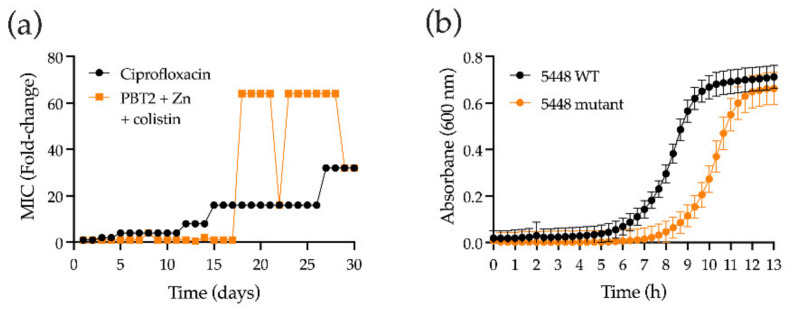
Resistance development against PBT2, zinc, and colistin. (**a**) Development of resistance by GAS strain 5448 during serial passage with colistin in the presence of sub-inhibitory concentrations of PBT2 and zinc (Zn) in CA-MHB supplemented with 2.5% (*v/v*) lysed horse blood. (**b**) Bacterial growth of GAS 5448 resistant mutant (PBT2 + Zn + colistin) compared to wild-type (WT) GAS 5448 in Todd-Hewitt broth supplemented with 1% (*w*/*v*) yeast extract. Growth curves are representative of three biological replicates.

We next investigated the possibility of resistance development to colistin in the presence of PBT2 + zinc for GAS strain 5448. During serial passage for a period of 30 days in the presence of sub-MIC concentrations of PBT2 + zinc and colistin, GAS strain 5448 demonstrated a 64-fold increase in the MIC to colistin (Figure 2a). A 32-fold increase in the MIC for the control antibiotic ciprofloxacin was also observed over the same time period (Figure 2a). However, increases in resistance to colistin in the presence of PBT2 + zinc imposed a fitness cost, as assessed by GAS 5448 growth rates in Todd–Hewitt broth supplemented with 1% (*w*/*v*) yeast extract (Figure 2b).

Whole genome sequencing (WGS) of GAS resistant to colistin in the presence of PBT2 + zinc identified the presence of six chromosomal differences when compared to the wild-type GAS 5448 reference genome (Supplementary Materials Table S1). For mutant GAS resistant to colistin in the presence of PBT2 + zinc, WGS identified a frameshift in *cadX*, a negative regulator of the CadDX efflux system that has been shown to confer resistance to cadmium and zinc toxicity in *S. salivarius* [24,25]. A secondary frameshift mutation was also observed in *mtsR*, a metal-dependent regulator of GAS manganese and iron import systems (e.g., MtsABC, *sia* operon) [26,27]. Although not extensively characterized for GAS, mutations in *cadX* may enable the constitutive expression of CadDX, potentially facilitating resistance to PBT2 + zinc-mediated potentiation of colistin in GAS strain 5448. Moreover, inactivation of *mtsR* has been previously shown to result in the constitutive transcription of the *sia* (streptococcal iron acquisition) operon in GAS, mediating the increased accumulation of intracellular iron [27]. Here, for GAS mutants resistant to colistin in the presence of PBT2 + zinc, *mtsR* inactivation and subsequent iron accumulation may enable an enhanced oxidative stress response via the iron-associated PerR (peroxide repressor)-regulated oxidative stress response system [28]. Collectively, these mutations, combined with the previously noted chromosomal differences, may explain the observed increases in resistance upon selection.

**Figure 3:**
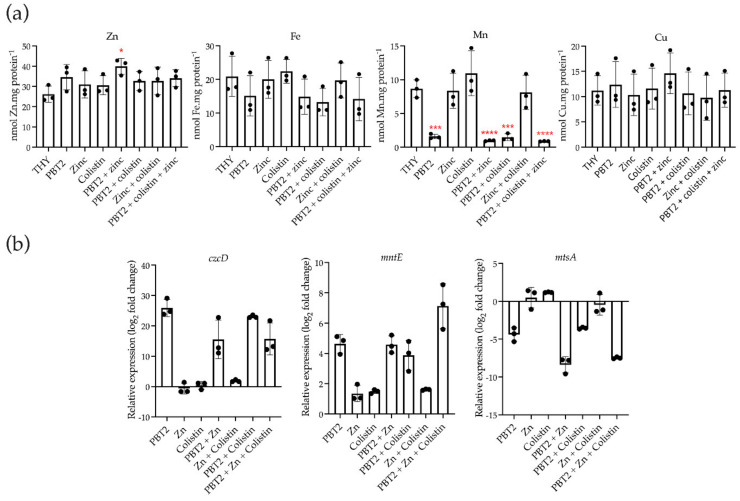
PBT2 dysregulates metal homeostasis in GAS strain 5448. (**a**) Intracellular zinc (Zn), manganese (Mn), iron (Fe), and copper (Cu) levels were assessed in GAS strain 5448 by inductively coupled plasma mass spectrometry. Bacteria were grown in Todd-Hewitt broth supplemented with 1% (*w/v*) yeast extract (THY) in the absence or presence of PBT2, zinc, and colistin. Error bars indicate standard error of the mean from three biological replicates, * *p* ≤ 0.05, *** *p* ≤ 0.001, **** *p* ≤ 0.0001, one-way ANOVA. (**b**) Transcript levels for *czcD*, *mntE*, and *mtsA* measured by quantitative real-time PCR. Log_2_-fold changes were calculated relative to untreated controls and normalized to the GAS 5448 reference gene *gyrA* using the ΔΔCt method. Error bars represent standard deviation of the mean of three biological replicates.

PBT2 facilitates the passive transport of zinc ions across biological membranes, independent of membrane protein-dependent transport pathways [6,29]. In this study, PBT2 treatment of GAS strain 5448 decreased the cellular content of manganese in a PBT2-dependent manner (Figure 3a). The impact of PBT2 on cellular manganese content was not affected by colistin and/or zinc. Furthermore, significant increases in the abundance of cellular zinc were observed only when bacteria were treated with PBT2 + zinc. For iron, although not significant, a general reduction in cellular iron content was observed when bacteria were treated with PBT2 alone or in combination with zinc and/or colistin. Cellular copper content was not affected by combinations of PBT2 with zinc and/or colistin (Figure 3a).

In the presence of zinc, PBT2 has been previously shown to induce intracellular zinc intoxication and manganese depletion in *S. uberis* and *S. pneumoniae* [30,31]. As such, quantitative real-time PCR was employed to examine potential transcriptional changes in the zinc-exporting cation diffusion facilitator gene *czcD; mntE*, a manganese-specific efflux pump paralog of *czcD* involved in oxidative stress; and *mtsA*, the gene encoding the solute-binding protein for the manganese-importing ATP-binding cassette transport pathway. Treatment with PBT2 in the presence and absence of zinc and/or colistin resulted in the upregulation of both *czcD* and *mntE,* and downregulation of *mtsA* (Figure 3b).

**Figure 4:**
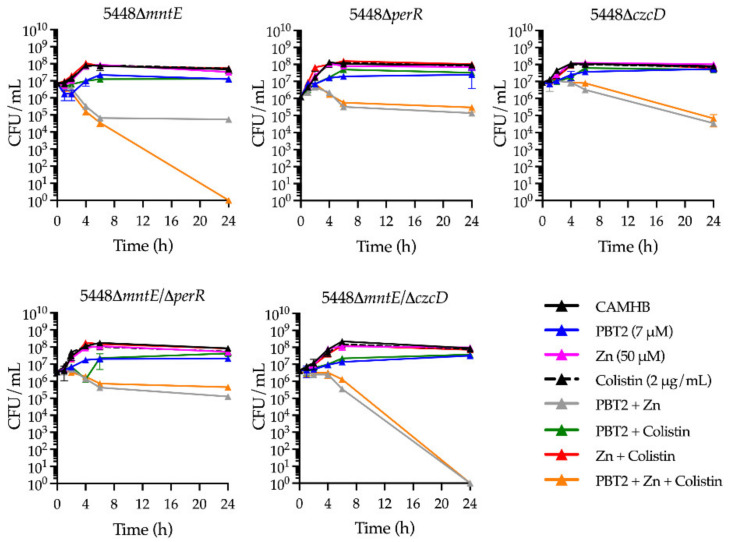
PBT2 + zinc + colistin drives an oxidative stress response in GAS strain 5448. Time-kill curves for GAS strain 5448 isogenic deletion mutants 5448Δ*mntE,* 5448Δ*perR*, 5448Δ*czcD,* and double deletion mutants 5448Δ*mntE–*Δ*perR* and 5448Δ*mntE–*Δ*czcD*. GAS mutants were cultured in Todd-Hewitt broth supplemented with 1% yeast extract (THY) and combinations of PBT2, zinc, and colistin. Error bars indicate standard deviation from three biological replicates. CFU, colony forming unit.

For GAS strain 5448, MntE maintains the homeostatic control of cytoplasmic manganese levels and indirectly influences the activity of the peroxide regulator, PerR [28]. Replacement of PerR-bound manganese by iron enables the transcription of oxidative stress response genes in GAS [28]. To investigate whether PBT2-mediated dysregulation of manganese and zinc cellular content was associated with a GAS oxidative stress response, we utilized the 5448 GAS isogenic deletion mutants 5448Δ*mntE,* 5448Δ*perR*, and 5448Δ*czcD,* and double deletion mutants 5448Δ*mntE–*Δ*perR* and 5448Δ*mntE–*Δ*czcD.* When compared to wild-type 5448 GAS, 5448Δ*mntE*, which constitutively represses PerR-regulated oxidative stress defense, demonstrated increased sensitivity to PBT2 + zinc + colistin exposure (Figure 1a and Figure 4). Conversely, 5448Δ*perR* demonstrated reduced sensitivity to colistin in the presence of PBT2 + zinc, suggesting that PBT2 + zinc + colistin leads to the production of reactive oxygen species (ROS) in GAS (Figure 4). This hypothesis was supported by the increased resistance of the 5448Δ*mntE–*Δ*perR* double deletion mutant (unable to efflux manganese yet adept to defend against oxidative stress) to PBT2 + zinc + colistin exposure (Figure 4). Moreover, a double deletion strain of *mntE* and *czcD*, 5448Δ*mntE–*Δ*czcD*, displayed increased sensitivity to both PBT2 + zinc and PBT2 + zinc + colistin, whereas the single *czcD* deletion mutant, 5448Δ*czcD*, only showed heightened sensitivity to PBT2 + zinc compared to wild-type GAS 5448 (Figure 1a and Figure 4). Collectively, these data suggest that PBT2 + zinc may trigger zinc toxicity and oxidative stress in 5448 GAS, and the addition of colistin induces further oxidative stress in the bacterium.

**Figure 5:**
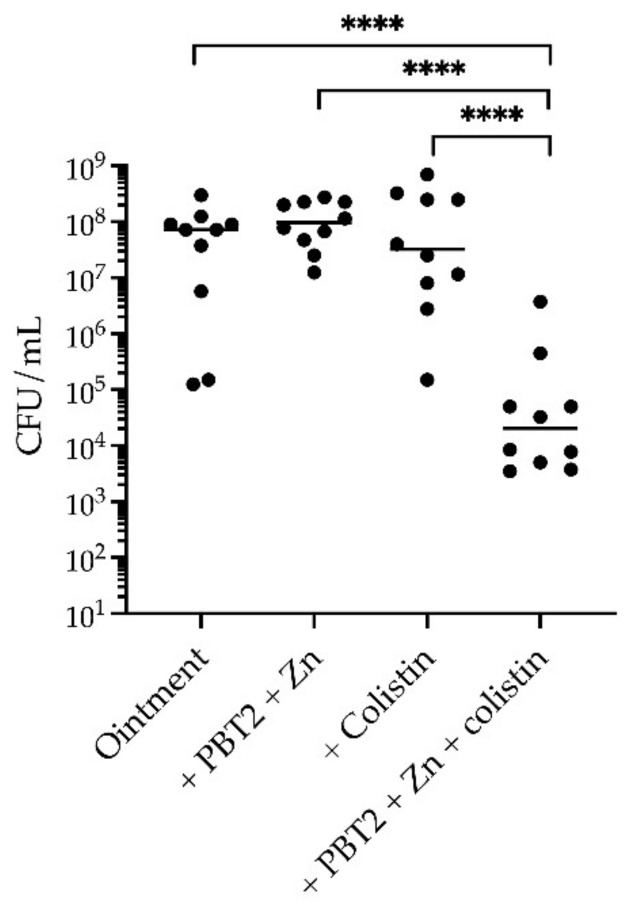
PBT2 and zinc breaks intrinsic resistance to colistin in a murine wound infection model. CFUs were recovered from cohorts of BALB/C mice (*n* = 10) 4 days after challenge with 2.77 × 10^6^ CFU of GAS strain 5448. Mice were treated twice daily with ointment only or ointment containing PBT2 (2 mM), ZnSO_4_ (25 mM), and colistin (20 µg/mL). Values for individual mice are plotted (**** *p* ≤ 0.0001, one-way ANOVA with Tukey multiple comparisons).

GAS is one of the most common causes of bacterial skin and soft tissue infections worldwide. To evaluate the efficacy of PBT2 + zinc in combination with colistin as a topical anti-infective therapy, we employed a murine model of wound infection. Neither colistin alone, nor PBT2 + zinc, significantly reduced the bacterial burden of GAS 5448 at the site of infection (Figure 5). However, when in combination, PBT2 + zinc and colistin significantly reduced the burden of GAS 5448 at the site of infection by >3-log_10_ (Figure 5). These data demonstrate that PBT2 + zinc can break intrinsic polymyxin resistance in GAS in vivo.

## 3. Discussion

Polymyxin antibiotics are commonly employed as last-resort treatment therapies for infections caused by MDR Gram-negative bacteria, particularly those caused by MDR *Pseudomonas aeruginosa* or carbapenemase-producing *Enterobacterales* [1]. Analogous to a detergent-like mode of action, polymyxin antibiotics induce a bactericidal effect in Gram-negative bacteria by binding to negatively charged lipid A moieties of the LPS. Although these interactions primarily lead to disruptions in both the outer- and cytoplasmic membrane structures [2,3], they have also been suggested to impair ribosome binding, respiration, cell division, and induce ROS in Gram-negative bacteria [32]. Regarding Gram-positive bacteria ubiquitously deficient in LPS, polymyxin antibiotics are inactive. Here, we demonstrate that the safe-for-human-use ionophore PBT2 potentiates the breaking of polymyxin resistance in intrinsically resistant Gram-positive human bacterial pathogens.

Previous work investigating the effects of PBT2 + zinc on *S. uberis* and *S. pneumoniae* has demonstrated that the combination of PBT2 and zinc leads to cellular manganese depletion and zinc intoxication [30,31]. Similarly, we show that cellular manganese levels in GAS strain 5448 are depleted in response to PBT2, whereas cellular zinc levels are raised in response to PBT2 + zinc treatment. Traditionally, manganese has been viewed as critical for defense against oxidative stress, i.e., metalation of superoxide dismutase with manganese for optimal function [26], and the protection of metalloenzymes against oxidative damage [33,34]. However, the regulation and subsequent export of cellular manganese in GAS by MntE has also been shown to play an anti-oxidative role. During infection, cytoplasmic manganese levels are regulated in GAS 5448 through MntE, enabling PerR to respond to neutrophil- and macrophage-stimulated peroxide stress [28]. In GAS, PerR predominantly exists as one of two complexes: (1) bound with iron, (2) bound with manganese. When bound with iron, PerR is highly sensitive to H_2_O_2_, leading to the upregulation of oxidative stress pathways. Opposingly, when manganese is reversibly coupled with PerR, a more stable complex is formed, resulting in the repression of the GAS oxidative stress defense system. PerR in GAS has also been shown to bind zinc, resulting in the repression of the GAS oxidative stress defense system [35]. Previous studies have demonstrated that PBT2 + zinc-treated *S. uberis* display H_2_O_2_ accumulation and an impairment in oxidative stress response systems. In line with this previous work [30], we propose that intracellular zinc accumulation, mediated by PBT2, may result in oxidative stress in GAS strain 5448. Furthermore, exposure to PBT2 + zinc in the presence of colistin may result in the additional accumulation of ROS, which may exceed the anti-oxidative capacity of the PerR system.

Exposure to PBT2 was shown to compromise the structural integrity of the GAS cell wall. The impact of altered manganese-to-zinc cellular ratios has previously been shown to perturb *S. pneumoniae* cell division [36]. This work revealed that the activity of the manganese-dependent protein phosphatase PhpP was directly influenced by the relative cellular abundance of zinc and manganese. Disruption of PhpP activity was shown to directly impact the cell division protein complexes MapZ and DivIVA, whose functions are regulated by PhpP/StkP-mediated phosphorylation [36]. Treatment with PBT2 and zinc induced oxidative stress, conferring a transcriptional response aligned with zinc intoxication. Although the molecular mechanism for PBT2 + zinc-driven polymyxin potentiation has yet to be defined, we speculate that PBT2 + zinc mediates oxidative stress in GAS by disrupting the dynamic interplay of zinc and manganese metal homeostasis during the PerR-regulated oxidative stress response, combined with perturbations in the activity of the cell division machinery. Furthermore, we also suggest that PBT2-mediated membrane perturbations may enable the entry of colistin into the bacterial cytoplasm, driving the potential impairment of ribosome binding, respiration, cell division, and induction of ROS formation [32]. Here, we have demonstrated that PBT2 + zinc can break intrinsic polymyxin resistance in Gram-positive pathogens. Although polymyxin antibiotics are not clinically employed for the treatment of Gram-positive infections, this study provides a platform from which to investigate the utility of PBT2 and next-generation polymyxin antibiotics with reduced toxicity profiles [1,6,37] for the treatment of MDR Gram-positive bacterial infections.

## 4. Materials and Methods

### 4.1. Materials

Colistin (catalogue no. C4461) and ciprofloxacin (catalogue no. 33434) were purchased from Sigma-Aldrich. PBT2 was produced by chemical synthesis [38], and the purity of the final product was confirmed to be >95% by 1H and 13C nuclear magnetic resonance, as previously described [23].

### 4.2. Construction of 5448ΔmntE–ΔczcD Mutant

GAS mutant 5448Δ*mntE–*Δ*czcD* was constructed by deletion replacement, as previously described [39]. Briefly*,* the 500 bp regions upstream of *mntE* in 5448Δ*czcD* were amplified using primers mntEKO-F1 and mntEKO-R1, respectively, whereas the 500 bp downstream regions were amplified using primers mntEKO-F2 and mntEKO-R2 (Table S2). The kanamycin cassette was amplified using primers kan-F and kan-R, and all three PCR fragments were joined together with the primers mntEKO-F1 and mntEKO-R2 (Table S2). The strain was confirmed by DNA sequencing (Australian Equine Genome Research Center, University of Queensland, Brisbane, Australia).

### 4.3. Bacterial Strains, Media, and Growth Conditions

GAS strains 5448 [21], NS178 [40], NS415 [41], NS179 [40], NS730 [40], BL16 [41], NS365 [42], NS192 [41], NS731 [41], NS473 [41]; *S. aureus* strains MRSA USA300 [23]; 25391-9848, 18542-6683, 19546-5182, 13127-8512, 27204-3593 (kindly provided by the Princess Alexandra Hospital, Brisbane); *Enterococcus* strains RBWH1 [23], GP_044 (kindly provided by Mark A.T. Blaskovich, University of Queensland); and 5448 GAS isogenic deletion mutants 5448Δ*mntE* [28]*,* 5448Δ*perR* [28]*,* 5448Δ*czcD* [39]*,* 5448Δ*mntE–*Δ*perR* [28], and 5448Δ*mntE–*Δ*czcD* (this study), were grown either in cation-adjusted Mueller–Hinton broth (CA-MHB) (Cat # 212322, Becton Dickson) supplemented with 2.5% (*v*/*v*) lysed horse blood (LHB), or in Todd–Hewitt medium supplemented with 1% (*w*/*v*) yeast extract (THY). Bacterial colony forming unit (CFU) enumeration was carried out on THY agar. Bacteria were routinely grown at 37 °C under static aerobic conditions.

### 4.4. Minimal Inhibitory Concentration (MIC) Determination

MICs and MIC breakpoints were determined by broth microdilution in accordance with CLSI and EUCAST guidelines [19,20]. Briefly, the precise concentration of PBT2 and zinc required to potentiate polymyxin sensitivity was determined by a series of two independent MIC experiments. Initial MIC experiments were undertaken with PBT2 alone. Secondary MIC experiments employed a constant concentration of PBT2 at ½ MIC, proceeded by the addition of zinc serially diluted across a 96-well plate. All MIC assays were carried out in biological triplicate.

### 4.5. Bacterial Time-Kill Assays

Bacteria were grown to an OD_600_ = 0.5 in THY, and treated with and without combinations of PBT2 (7 µM), ZnSO_4_ (50 µM), and colistin (2 µg/mL) for 24 h at 37 °C. To determine the rate of bacterial killing, aliquots of bacterial suspension were removed at 0, 1, 2, 4, 6, and 24 h, serially diluted in PBS, and plated onto THY agar plates. Time-kill assays were performed in biological triplicate.

### 4.6. Scanning Electron Microscopy (SEM)

SEM studies were undertaken at the Center for Microscopy and Microanalysis at the University of Queensland. GAS strain 5448 was cultured in THY to OD_600_ = 0.5, and treated in the absence and presence of PBT2 (7 µM), zinc (50 µM), and colistin (2 µg/mL) for 24 h at 37 °C. Bacteria were washed twice with PBS preceding glutaraldehyde fixation. Samples were then dehydrated, assisted with a Pelco BioWave regimen, via a series of ethanol treatments (30–100% EtOH), one treatment with 100% EtOH/hexamethyldisilazane (HMDS; 1:1) and, finally, two treatments with 100% HMDS. Samples were applied to coverslips coated with poly-*L*-lysine (1 mg/mL) before being air-dried for 2 h. Coverslips were attached to 13 mm SEM-stubs with double-sided carbon tabs, plasma-cleaned for 10 min in an Evactron De-Contaminator (XEI Scientific), and coated with two layers of platinum (first layer 0° angle from above, second layer 45° angle from above) using a Turbomolecular pumped coater (Quorum Tech) following manufacturer’s instructions. Samples were imaged in a JEOl JSM 7100F or JEOl JSM 7800F field emission SEM at an accelerating voltage of 1–3 kV and at a working distance of 10 mm.

### 4.7. Resistance Development Assays 

The development of resistance to colistin in the presence of PBT2 and zinc was undertaken as previously described [6,23]. Briefly, GAS strain 5448 was sequentially passaged in CA-MHB + 2.5% LHB containing sub-inhibitory concentrations of PBT2 + Zn + colistin over 30 days at 37 °C. As a control for resistance development, the antibiotic ciprofloxacin was used. Initially, the MIC for PBT2 + Zn with or without antibiotic was determined by broth microdilution following CLSI guidelines in a microtiter plate [19]. The highest antibiotic or PBT2 + Zn + antibiotic concentration that still showed growth after overnight incubation was further diluted 1:250 (in CA-MHB + 2.5% LHB) into a new microtiter plate containing two-fold dilutions of antibiotic or PBT2 + Zn + antibiotic. This procedure was repeated for 30 days.

### 4.8. Whole Genome Sequencing Analysis

DNA was extracted from overnight cultures of wild-type and mutant GAS isolates resistant to colistin in the presence of PBT2 + Zn using the Wizard Plus SV Minipreps DNA purification System (Promega) as per the manufacturer’s instructions. Genomic DNA was sequenced on NovaSeq 6000 using 150 base paired-end reads from Illumina Nextera XT libraries at Victorian Clinical Genetics Services (Parkville, VIC, Australia). Sequences of day 0 and day 30 were mapped to the M1GAS strain 5448 (Genbank reference sequence GCF_900619585.1) using Breseq v0.35.1 [43].

### 4.9. Growth Analysis

Overnight cultures of wild-type GAS strain 5448, and the GAS strain 5448 resistant to colistin in the presence of PBT2 + Zn, were standardized to OD_600nm_ = 0.01 in THY medium. Bacteria were grown in a 96-well plate at a final volume of 200 μL and measured at 600 nm using a FLUOstar Omega microplate reader (BMG Labtech) at 37 °C without shaking. Growth assays were performed in biological triplicates and measured in technical replicates of twelve.

### 4.10. Inductively Coupled Plasma Mass Spectrometry (ICP-MS)

From overnight cultures, GAS strain 5448 was grown to an OD_600_ = 0.5 in THY. Cells were then treated with combinations of PBT2 (7 µM), zinc (50 µM), and colistin (2 µg/mL) for 30 min at 37 °C. Cells were harvested, processed, and analyzed using an Agilent 8900 ICP-QQQ as previously described [6,23].

### 4.11. RNA Isolation 

RNA was isolated using the RNeasy kit (Qiagen) as previously described [6,23]. Briefly, bacteria were grown to mid-log phase in CA-MHB (+2.5% LHB) in the presence or absence of PBT2, zinc, and/or colistin. The samples were centrifuged at 4000× *g* for 10 min at 4 °C to form a pellet. The dry pellet was processed according to the manufacturer’s recommendations (Qiagen RNeasy mini kit) and eluted in 80 μL of nuclease-free water. To ensure complete removal of DNA, the RNA was then further purified using the TURBO DNA-free kit (Thermo Fisher Scientific, Waltham, MA, USA) according to the manufacturer’s instructions.

### 4.12. Quantitative Real-Time PCR 

Genes associated with zinc and manganese homeostasis were selected for quantitative real-time PCR analysis. Quantitative real-time PCR was carried out as previously described [6]. Relative gene expression was calculated by the ΔΔCT method using *gyrA* as the reference gene for GAS strain 5448. All experiments were conducted in biological triplicates and measured in technical triplicates. Primers used for quantitative real-time PCR are provided in Table S2.

### 4.13. Murine Wound Infection Model

For wound infection, 7-week-old female BALB/C mice were prepared, anesthetized, and subjected to superficial scarification as previously described [23]. For infection, GAS strain 5448 was cultured to mid-log phase in THY and 2.77 × 10^6^ colony forming units (CFU) of bacteria were applied onto the scarified tissue in a final volume of 10 µL. Mice cohorts (*n* = 10) were treated with either topical carrier ointment only (Pharmacy Choice aqueous cream), or topical ointment containing combinations of PBT2 (2 mM), ZnSO_4_ (25 mM), and colistin (25 µg/mL). Treatments were administered twice daily for 4 days. At 4 days post-infection, mice were euthanized, the skin was excised, and bacteria were enumerated on THY agar plates supplemented with 10 μg/mL neomycin. Statistical differences in CFU were determined by a one-way ANOVA, with *p* < 0.05 considered statistically significant (GraphPad Prism 9).

### 4.14. Ethics

Animal experiments were performed according to the Australian code of practice for the care and use of animals for scientific purposes. Permission was obtained from the University of Queensland ethics committee (SCMB/AIBN/144/17).

### 4.15. Statistical Analysis

All experiments were undertaken either in technical duplicate or triplicate, with no-less than three biological replicates. Data are presented as means ± SEM. Bacterial infection data from in vivo studies are presented as geometric means. To compare means between more than two groups, a one-way ANOVA with a post hoc (Tukey) test was conducted. Statistical analysis was performed using GraphPad Prism software v9. *p* < 0.05 was considered to be statistically significant.

## Figures and Tables

**Table 1:** The combination of PBT2 and zinc breaks intrinsic polymyxin resistance in Gram-positive bacterial pathogens. Resistance to colistin was assessed for multiple strains of GAS, *S. aureus*, and *Enterococcus*. MIC assays were undertaken in the absence (untreated) or presence of PBT2, zinc, or PBT2 and zinc. MIC values highlighted in bold indicate an antibiotic susceptible breakpoint (≤2 µg/mL) in accordance with EUCAST guidelines for antimicrobial sensitivity testing of colistin against Gram-negative bacteria. Data represents the mean of three biological replicates. MRSA, methicillin resistant *S. aureus*; VRE, vancomycin-resistant *Enterococcus*.

Strain	Concentration (μM)		Colistin MIC (μg/mL)			
	PBT2	ZnSO_4_	CA-MHB	+PBT2	+ZnSO_4_	+PBT2 + ZnSO_4_
**Group A *Streptococcus***						
5448	7	50	>128	32–64	>128	**≤0.125–0.25**
NS178	3.75	64	>128	16	>128	**1**
NS415	3.25	64	>128	32	>128	**0.25**
NS179	2.25	64	>128	32	>128	**0.5**
NS730	3.25	64	>128	16	>128	**≤0.125**
BL16	3.25	64	>128	16	>128	**≤0.125**
NS365	3.25	64	>128	16	>128	**≤0.125**
NS192	3.25	64	>128	32	>128	**0.25**
NS731	3.75	64	>128	32	>128	**1**
NS473	3.25	64	>128	32	>128	**0.25**
***S. aureus***						
USA300 (MRSA)	8	50	>128	32	>128	**2**
25391-9848	2	60	>128	64	>128	**2**
18542-6683	2.5	60	>128	64	>128	**2**
19546-5182	2	60	>128	32	>128	**0.5**
13127-8512	2.5	60	>128	64	>128	**1**
27204-3593	3	60	>128	32	>128	**0.5**
***E. faecium***						
RBWH1 (VRE)	1.75	128	>128	16	>128	**≤0.125–0.25**
GP_044 (VRE)	4	64	>128	128	>128	**2**

## Data Availability

Genome data has been deposited to NCBI under BioProject PRJNA786398. Raw Illumina sequence read data has been deposited to the sequence read archive (SRA) under the accessions SRR17138560 (GAS 5448 WT) and SRR17138559 (GAS 5448 mutant).

## Supplementary Materials

The following are available online at https://www.mdpi.com/article/10.3390/antibiotics11040449/s1, Figure S1: Scanning electron microscopy images of GAS strain 5448 grown in THY media, or in THY media supplemented with combinations of PBT2 (7 µM), zinc (50 µM), and colistin (2 µg/mL) for 24 h at 37 °C, Table S1: Mutant chromosomal differences as identified by Illumina whole genome sequencing, Table S2: Primers used in this study.
