# Supplementary material for: Neurodegenerative Disease Treatment Drug PBT2 Breaks Intrinsic Polymyxin Resistance in Gram-Positive Bacteria
Source: Antibiotics (Basel). 2022 Mar 25;11(4):449. doi: 10.3390/antibiotics11040449 (PMC9027797; doi:10.3390/antibiotics11040449)
Supplement: Supplementary file 1 [file antibiotics-11-00449-s001.zip › Supplementary material/Supplementary Material.pdf]

Supplementary Material 1:

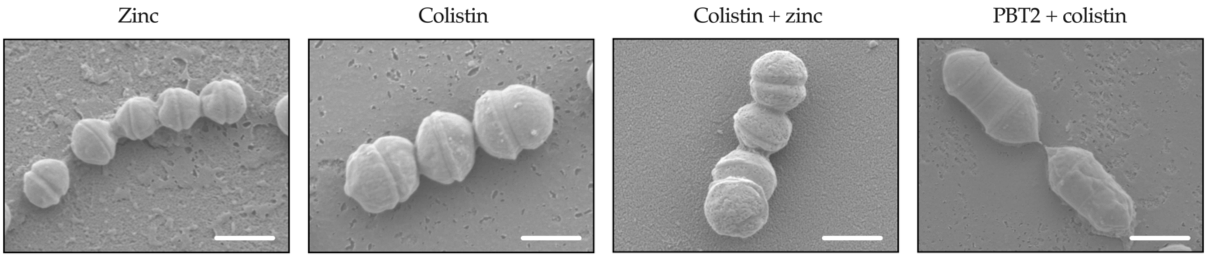

Figure S1. Scanning electron microscopy images of GAS strain 5448 grown in THY media, or in THY media supplemented with combinations of PBT2 (7 μM), zinc (50 μM), and colistin (2 μg/mL) for 24 h at 37°C. White scale bars = 1 μm. Arrows indicate membrane indentations and membrane ruffling.

Supplementary Material 2:

Table S1. GAS 5448. Mutant chromosomal differences as identified by Illumina whole genome sequencing.

**Locus_tag**	**Gene; Product**	**Position**	**Mutation**
SP5448_08615	*pabB;* aminodeoxychorismate synthase component I	205,511	$(TGGCTG)_{1\rightarrow 2}$
SP5448_08450	*pnp ;* polyribonucleotide nucleotidyltransferase	247,553	+GCG
SP5448_07680	*mtsR;* metal dependant transcriptional regulator	390,289	Δ1 bp
SP5448_06655	*atpA;* F0F1 ATP synthase subunit alpha	596,049	C→G
SP5448_06005	*parC;* DNA topoisomerase IV subunit A	734,072	$(TCCTTC)_{1\rightarrow 2}$
SP5448_09200	*cadX;* Cd^{2+} Zn^{2+}-sensing metalloregulatory transcriptional regulator	1,777,811	$(T)_{7\rightarrow 6}$

Supplementary Material 3:

Table S2. Primers used in this study.

**GAS 5448**		
Deletion mutation and reverse complementation constructs		
Primer	Sequence 5′→3′	
mntEKO-F1	CCCGGCTCGAGAAACCAATGAACCCGTGGAG	
mntEKO-R1	CAAAGTTGGCGTATAACATATGCAATCGGATCCTGTGTCA	
mntEKO-F2	TGATATTCTCATTTTAGCCATTGCAATCGGATCCTGTGTCA	
mntEKO-R2	CGCGCGAAGCTTTTTACAGCCAACAGACCGTG	
kan-F	ATGGCTAAAATGAGAATATCA	
kan-R	CTAAAACAATTCATCCAGTAA	
Quantitative real-time PCR		
Gene	Primer direction	Sequence 5′→3′
czcD	Fwd	ACCATCCATCGACCAAACAT
czcD	Rev	ATTGCACTTTGCACCATGAA
mntE	Fwd	CCATGATTCCCGAAGAAGAG
mntE	Rev	CATTCGCATCAATCAGTTGG
mtsA	Fwd	CAAACAATTGATTGCAAAGGATCC
mtsA	Rev	GTCTAGTTTTTCCAATTTAGCCACA
gyrA	Fwd	GAAGTGATCCCTGGACCTGA
gyrA	Rev	CCCGACCTGTTTGAGTTGTT
